# Marine Gelatin-Methacryloyl-Based Hydrogels as Cell Templates for Cartilage Tissue Engineering

**DOI:** 10.3390/polym15071674

**Published:** 2023-03-28

**Authors:** Inês Machado, Catarina F. Marques, Eva Martins, Ana L. Alves, Rui L. Reis, Tiago H. Silva

**Affiliations:** 1 3B’s Research Group, I3B’s—Research Institute on Biomaterials, Biodegradables and Biomimetics, University of Minho, Headquarters of the European Institute of Excellence on Tissue Engineering and Regenerative Medicine, AvePark, Parque de Ciência e Tecnologia, Zona Industrial da Gandra, Barco, 4805-017 Guimarães, Portugal; 2 ICVS/3B’s—PT Government Associate Laboratory, 4806-909 Braga/Guimarães, Portugal

**Keywords:** marine biomaterials, fish gelatin, GelMA, cartilage, chondrocytes, methacrylation, photocrosslinking

## Abstract

Marine-origin gelatin has been increasingly used as a safe alternative to bovine and porcine ones due to their structural similarity, avoiding the health-related problems and sociocultural concerns associated with using mammalian-origin materials. Another benefit of marine-origin gelatin is that it can be produced from fish processing-products enabling high production at low cost. Recent studies have demonstrated the excellent capacity of gelatin-methacryloyl (GelMA)-based hydrogels in a wide range of biomedical applications due to their suitable biological properties and tunable physical characteristics, such as tissue engineering applications, including the engineering of cartilage. In this study, fish gelatin was obtained from Greenland halibut skins by an acidic extraction method and further functionalized by methacrylation using methacrylic anhydride, developing a photosensitive gelatin-methacryloyl (GelMA) with a degree of functionalization of 58%. The produced marine GelMA allowed the fabrication of photo-crosslinked hydrogels by incorporating a photoinitiator and UV light exposure. To improve the biological performance, GelMA was combined with two glycosaminoglycans (GAGs): hyaluronic acid (HA) and chondroitin sulfate (CS). GAGs methacrylation reaction was necessary, rendering methacrylated HA (HAMA) and methacrylated CS (CSMA). Three different concentrations of GelMA were combined with CSMA and HAMA at different ratios to produce biomechanically stable hydrogels with tunable physicochemical features. The 20% (*w*/*v*) GelMA-based hydrogels produced in this work were tested as a matrix for chondrocyte culture for cartilage tissue engineering with formulations containing both HAMA and CSMA showing improved cell viability. The obtained results suggest these hybrid hydrogels be used as promising biomaterials for cartilage tissue engineering applications.

## 1. Introduction

Articular cartilage (AC) is a viscoelastic connective tissue that comprises a cellular fraction consisting of articular chondrocytes and an extracellular matrix (ECM) that is mainly composed of matrix-associated water, collagens, glycosaminoglycans (GAGs) and other glycoproteins, which provide the tissue with the mechanical properties required to absorb impacts and withstand significant loads. However, cartilage is frequently damaged due to trauma, disease, or even wear and has a limited self-regenerative capacity due to the lack of vasculature, responsible for nutrition supply, meaning that untreated pathologies can lead to the development of osteoarthritis, causing pain, loss of mobility and significant economic burden [1,2].

Surgical interventions using autologous cell populations are the standard procedure for addressing cartilage malformation. Although these surgeries can effectively promote the formation of new tissue, post-surgery histological exams often reveal the development of mechanically inferior fibrocartilage, which increases the risk of implant failure [3]. It is mostly due to the chondrocyte’s dedifferentiation during expansion in two-dimensional cultures. This process is characterized by the gradual loss of molecular markers that characterize a differentiated chondrocyte. As dedifferentiation advances, expression of ECM molecules as GAGs and type II collagen is lost, while simultaneously, the cells acquire an increased fibroblastic phenotype [2]. Conventional two-dimensional (2D) monolayer cultures are observed as unsuitable, as monolayer expansion of articular chondrocytes does not support the chondrogenic phenotype through passaging. As an alternative, it is well accepted that high cell density three-dimensional (3D) cultures favor the preservation of the chondrocyte phenotype [4]. Therefore, articular cartilage repair using a tissue engineering approach has drawn growing attention.

In tissue engineering strategies, scaffolding materials, acting as a temporary extracellular matrix, must provide both a support structure for the cells and, at the same time, a biochemical niche that allows the cells to deposit their specific extracellular matrix [5]. Hydrogels, as 3D hydrophilic scaffolds, are a suitable platform for maintaining chondrocyte phenotype and promoting the formation of AC-like tissue by homogenously distributing cells [6]. Various materials have been tested as components of these hydrogels, in which cells are seeded before implantation. In recent years, gelatin-methacryloyl (GelMA) hydrogels have been in the spotlight of the tissue engineering field, as a series of different gels can be obtained by controlling the production parameters. Furthermore, GelMA can be manufactured through several methods, and each method produces different shapes of GelMA for different purposes. It makes GelMA a promising and inexpensive material for biological applications with a higher melting point than gelatin alone [2,7]. Although dedifferentiation of chondrocytes can occur within GelMA hydrogel of low stiffness (1.5 kPa) [8], in stiffer GelMA hydrogels (approximately 30 kPa), chondrogenic redifferentiation occurs, both in vitro [9] and in vivo [10]. GelMA can be produced by chemical modification of gelatin, allowing hydrogels to be covalently crosslinked in the presence of a photoinitiator and UV light [11]. Nevertheless, gelatin is mostly produced from porcine and bovine tissues, raising religious and ethical issues associated with the use of mammal-origin materials and concerns regarding the risk of zoonosis, from which marine gelatin derived from cold-water fish has been receiving increasing attention [12,13]. In the fish processing industry, up to three-quarters of the raw materials are wasted as by-products, increasing the pressure for effective valorization strategies [14,15,16], with countless works testing different fish by-products to produce gelatins [17], promising a safe and low-cost alternative solution, with inherent bioactivity and physicochemical tunability [18].

Modification of the GelMA scaffold materials by glycosaminoglycans such as hyaluronic acid and chondroitin sulphate, which are important components of ECM, was introduced to enhance cells’ migration, adhesion, proliferation, and differentiation and to promote the preservation of the differentiated states of the cells, as compared to GelMA alone [19]. Hyaluronic acid (HA) possesses high-capacity lubrication, water-sorption and water retention. For human articular chondrocyte cultures, HA can be functionalized with methacrylate groups by reaction with methacrylic anhydride to yield HAMA, which enables the covalent and stable incorporation of HA into the hydrogel matrix and is shown to enhance chondrogenic cell differentiation, accumulation of cartilaginous ECM and mechanical properties in articular cartilage tissue engineering applications when compared to GelMA alone [20]. Chondroitin sulfate (CS) is a sulfated glycosaminoglycan and an important structural component of cartilage that provides resistance to compression. It can also be functionalized with methacrylate groups by reaction with methacrylic anhydride to yield CSMA [21]. Levett et al. [1] showed that incorporating CSMA enhanced some aspects of chondrocyte redifferentiation, though to a lesser extent than HAMA. However, the greatest increase in stiffness was seen in GelMA, HAMA and CSMA constructs, suggesting that there may be advantages to including both GAGs in the hydrogels [1].

This work aimed to investigate the production of photo-crosslinked fish gelatin-methacryloyl-based hydrogels and assess their potential as templates for the 3D culture of chondrocytes envisaging, ultimately, cartilage regeneration. The first step was to extract and characterize marine gelatin from the skin of Greenland Halibut, a by-product from the industrial processing of this fish species for food. After that, the fish gelatin was methacrylated, and hydrogels were produced. Aiming to improve chondrocyte performance in GelMA-based hydrogels, glycosaminoglycans, particularly CS and HA, were incorporated into GelMA hydrogels separately or together. To allow the covalent and stable incorporation of these GAGs into GelMA hydrogels and to make photocrosslinking possible, methacrylated CS and HA were used (CSMA and HAMA, respectively). Then the physicochemical properties of the different hydrogels’ formulations were performed and compared, namely regarding water uptake and enzymatic degradability. Finally, the performance of the hydrogels as cartilage tissue engineering scaffolds was assessed in vitro by a culture of chondrocyte-like cells for up to 14 days and evaluating their viability, proliferation, and morphology.

## 2. Materials and Methods

### 2.1. Materials

Fish gelatin was extracted from the skin of Greenland Halibut (*Reinhardtius hippoglossoides*), a by-product of industrial fish processing for food, as described below. Standard commercial gelatin type A and B, chondroitin sulfate sodium salt from shark cartilage, hyaluronic acid sodium salt from *Streptococcus equi*, methacrylic anhydride and 2-Hydroxy-4′-(2-hydroxyethoxy)-2-methylpropiophenone were purchased from Sigma Aldrich, St. Louis, MO, USA and used as received.

### 2.2. Gelatin Extraction

The gelatin was extracted by an acidic extraction method based on the protocol described by Arnesen and Gildberg [22]. For that, 500 g of Greenland halibut skins were washed in cold distilled water, cut into small pieces, and then soaked two times in cold 0.04 M NaOH (1.65 L) for 30 min. After washing out the NaOH with cold distilled water, two successive incubations in acid solutions were performed for 30 min each, first in 0.12 M sulfuric acid (1.65 L) and then in 0.005 M citric acid (1.65 L). Next, the acid solutions were drained, and the fish skins were washed again with cold distilled water. After that, a two-step gelatin extraction was performed by gentle stirring in 5 L water at 55 °C for 2 h and then at 65 °C for another 2 h. Subsequently, solubilized gelatin was separated from residual skin fragments by 4 different filtration stages: first through a mesh strainer, second through a 250 µm mesh nylon filter, third with a Whatman No. 4, and fourth with a GF/C filter. Finally, the gelatin extract was concentrated in a rotavapor (40 °C) before drying as a thin film in an oven at 50 °C and was stored as fragments at RT until further use.

The extraction yield of gelatin was calculated from the dry weight (g) of the extracted gelatin and wet weight (g) of the by-product as follows (Equation (1)) [22]:

(1) $$\mathrm{Yield}=\frac{Dry\;weight\;of\;extracted\;gelatin}{Wet\;weight\;of\;by\text{-}products}\;\times \;100$$

### 2.3. Functionalization of Halibut Gelatin

**Figure 1:**
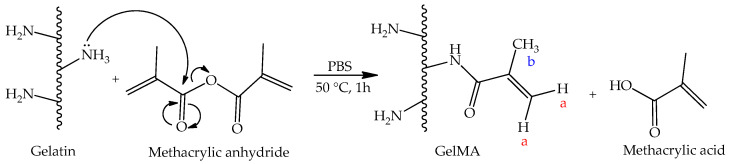
The reaction of gelatin functionalization with methacrylic anhydride.

To obtain marine-origin gelatin-methacryloyl (Figure 1), the extracted gelatin was functionalized with methacrylic anhydride (MA) to incorporate photo-sensible methacryloyl groups in the gelatin backbone, according to Loessner et al. [11]. Briefly, the gelatin was soaked to a final concentration of 10% (*w*/*v*) in PBS for 60 min at 60 °C. While stirred vigorously, 0.6 g of methacrylic anhydride was slowly added per 1 g of dissolved gelatin, and the acylation reaction was performed for 60 min. After the reaction period, the unreacted methacrylic anhydride was removed by centrifugation at 3500× *g* for 3 min at RT. The supernatant solution was diluted with two volumes of preheated (40 °C) PBS and dialyzed for 5–7 days until the GelMA solution appeared clear. The odor of residual methacrylic anhydride or methacrylic acid by-product is no longer noticeable. The pH of the GelMA solution was adjusted to 7.4 using 1 M NaHCO_3_ and filter-sterilized using 0.2 μm syringe filter units. After that, GelMA was freeze-dried and stored at −20 °C until further use.

### 2.4. Determination of the Degree of Functionalization (DoF) of GelMA

To determine the DoF of GelMA, the method of ^1^H NMR was used. For that, 20 mg of GelMA and non-functionalized gelatin was completely dissolved in 1 mL of deuterium oxide. The DoF was calculated from the lysine methylene proton of GelMA, and the lysine methylene proton of gelatin as follows (Equation (2)) [23,24,25,26]:

(2) $$\%\;\mathrm{DoF}=1-\frac{area\;of\;lysine\;proton\;signal\;in\;GelMA}{area\;of\;lysine\;signal\;in\;Gelatin}\;\times \;100$$

### 2.5. Functionalization of Hyaluronic Acid and Chondroitin Sulfate

Hyaluronic acid and chondroitin sulfate were also reacted with methacrylic anhydride using protocols based on published methods [1,27]. Briefly, hyaluronic acid and chondroitin sulphate were dissolved in PBS at 1% and 25% (*w*/*v*). CS and HA were reacted with methacrylic anhydride for 24 h at 4 °C, with the pH regularly adjusted to 8 with NaOH. For HA and CS, the molar excess of methacrylic anhydride over the hydroxyl groups was 5- and 8-fold, respectively. After the reaction, insoluble methacrylic anhydride was removed by dialysis against ultrapure water to remove the remaining unreacted methacrylic anhydride and methacrylic acid by-product. The pH of the dialyzed polymer solutions was adjusted to 7.4, and after that, they were freeze-dried and stored at −20 °C until further use. To confirm that functionalization occurred was used ^1^H NMR analysis.

### 2.6. Physical and Chemical Characterization of Biopolymers

#### 2.6.1. FTIR Analysis

To evaluate the presence of gelatin’s characteristic chemical bonds and groups, freeze-dried gelatin samples were analyzed by Fourier-transform infrared (FTIR) spectroscopy (IRPrestige 21—Shimadzu, Kyoto, Japan) in attenuated total reflectance (ATR) mode between 4000 and 500 cm^−1^ with a resolution of 2 cm^−1^, with each spectrum being the average of 32 scans.

#### 2.6.2. CD Analysis

To evaluate the protein conformation of the extracted gelatin, circular dichroism (CD) analysis was performed (J1500 CD spectrometer, JASCO, Tokyo, Japan) using a quartz cylindrical cuvette with a path length of 2 mm. Gelatin samples were dissolved at 0.1 mg/mL, and 600 µL aliquots were added to the cuvette. CD spectra (average of three scans) were obtained from 180 to 240 nm by a scan rate of 50 nm/min at 37 °C.

### 2.7. Fabrication of GelMA Hydrogels

The lyophilized GelMA was dissolved in PBS at three different concentrations and later combined with CSMA and/or HAMA at different ratios, according to the formulations depicted in Table 1. The photoinitiator 2-hydroxy-4′-(2-hydroxyethoxy)-2-methylpropiophenone was then added in a concentration of 0.05% (*w*/*v*). After proper dissolution, 80 µL aliquots of the resulting solution were placed into silicone mold wells (with 6 mm diameter) and exposed to ultraviolet light (366 nm, UV lamp Triwood 6/36; Bresciani srl., Italy, 0.120 J/cm^2^) at RT for 15 min to promote photocrosslinking [11].

### 2.8. Characterization of GelMA Hydrogels

#### 2.8.1. Water Absorption Capacity

To determine the water absorption capacity of hydrogels, five samples (*n* = 5) from each formulation (A–L) were used. First, all samples were weighed (*W_i_*), then immersed in PBS solution, and incubated in an oven at 37 °C. After a certain period of time (0 h, 30 min, 1 h, 24 h, 48 h, 72 h, 7 days, 14 days, and 21 days), the samples were taken out of the solution and gently soaked between two filter papers to remove excess of liquid and weighted immediately (*W_t_*). Then, the percentage of swelling due to water uptake was calculated using the following formula [28]:

(3) $$\%\;\mathrm{Swelling}=\frac{Wt\;-\;Wi}{Wi}\;\times \;100$$

#### 2.8.2. Enzymatic Degradation Capacity

To characterize the enzymatic degradation properties of hydrogels, three samples (*n* = 3) of each formulation (A–L) were weighted (*Wi*) and immersed in PBS containing 2 µg/mL collagenase A (Roche Diagnostics) and incubated at 37 °C [29,30]. The samples were weighed (*Wt*) at different time points (1 h, 2 h, 3 h, 6 h, 12 h, 1 day, 2 days, 3 days, 7 days, 14 days, and 21 days). At each weighing, the samples were removed from the container, rinsed with distilled water, and then dried to constant mass at room temperature in a desiccator. The percentage of mass loss was determined by the following formula [31]:

(4) $$\%\;\mathrm{Mass}\;\mathrm{Loss}=\frac{Wi\;-\;Wt}{Wi}\;\times \;100$$

### 2.9. Biological Assessment

#### 2.9.1. Cell Culture

For the biological assessment, the hydrogels produced with 20% (*w*/*v*) GelMA solution were used. Freshly prepared GelMA-based hydrogels were washed 3 times with PBS solution to remove the excess of 2-Hydroxy-4′-(2-hydroxyethoxy)-2-methylpropiophenone and placed in 48-wells tissue culture plates. Cells from the chondrogenic cell line ATDC-5 (ECACC, UK) at passage 14 were seeded on top of hydrogels at a concentration of 200,000 cells/hydrogel. The cultures were maintained during 14 days in DMEM: F12 medium (Invitrogen, Waltham, MA, USA) supplemented with 10% FBS (Invitrogen, Waltham, MA, USA) and 1% antibiotic/antimycotic (Invitrogen, Waltham, MA, USA) at 37 °C and 5% of CO_2_, and the medium was replaced every 2–3 days.

#### 2.9.2. Live/Dead Assay

After 1, 7 and 14 days in culture, the cell viability was evaluated with a live/dead viability assay. Briefly, the hydrogels with seeded cells were incubated in dark conditions for 30 min at 37 °C in a culture medium containing calcein (CA) (1:500), which stained the cytoplasm of live cells and generated green fluorescence, and propidium iodide (PI) (1:1000), which binds to DNA and fluoresces red. After incubation, samples were observed in a Leica SP8 Inverted Confocal Microscope.

#### 2.9.3. Analysis of Cell Metabolic Activity and Proliferation

The metabolic activity of cells cultured in the GelMA-based hydrogels were measured using ALAMAR BLUE^®^ (AbD Serotec, Oxford, UK). At each time, the hydrogels were incubated with 10% (*v*/*v*) of Alamar Blue in DMEM-F12 culture medium for 3 h protected from light, at 37 °C and with 5% CO_2_. Afterwards, 100 μL of supernatant was transferred from each well in triplicate to a new 96-well tissue cultures plate. Fluorescence intensity was read at 530/20 nm (excitation) and 590/35 nm (emission) using a microplate reader (Synergy HT, Bio-Tek, Winooski, VT, USA). Alamar Blue in the medium was used as a blank. 

To quantify cell proliferation, total double-stranded DNA (dsDNA) was assessed. First, at each time point, a lysed cell suspension was obtained after osmotic and thermal shocks by incubating them for 1 h at 37 °C in ultrapure water, followed by freezing at −80 °C, thawed and sonicated for 15 min. This cell lysate was used for dsDNA quantification using the Quant-iT PicoGreen dsDNA kit (Molecular Probes, Invitrogen), according to the manufacturer’s instructions. Then, fluorescence was read in a microplate reader at an excitation of 485/20 nm, and emission of 528/20 nm and RFUs were converted into ng/mL using a standard curve of DNA in the range of 1–2 μg/mL.

#### 2.9.4. SEM Analysis

For cell morphology and cell distribution analysis, the GelMA-based hydrogels were analyzed by Scanning Electron Microscopy (SEM, model JSM-6010 LV, JEOL, Tokyo, Japan). At different time points (1, 7 and 14 days in culture), the hydrogels were washed in PBS and fixed in 4% (*w*/*v*) paraformaldehyde in PBS for 20 min RT, followed by dehydration with graded ethanol series (60%, 70%, 80%, 90%, and 99%) for 10 min in each solution and overnight in hexamethyldisilazane (Sigma-Aldrich). The samples were sputter-coated with an electrically conducting layer of platinum, and the micrographs were obtained at 10 kV at 100×, 500× and 1500× magnifications.

#### 2.9.5. Statistical Analysis

Statistical analysis of the biological tests was made using GraphPad Prism 7.0 software (GraphPad Software, La Jolla, CA, USA), by a non-parametric Kruskal- Wallis test followed by Dunn’s test for multiple comparisons was used. The significance level was set to * *p* < 0.05, ** *p* < 0.01, *** *p* < 0.001, **** *p* < 0.0001.

## 3. Results and Discussion

### 3.1. Gelatin Extraction

A large part of a fish’s weight, such as skins, scales, and fish bones, is discarded due to industrial processing, posing as a valuable source of raw materials [32,33]. Among marine sources, fish skins are often chosen to extract gelatin, presenting advantages like being available at a large scale, having no risk of transmitting diseases, and having no religious constraints. Halibut (*Reinhardtius hippoglossoides*) skins were selected as raw material for fish gelatin extraction since this is a very industrially processed species, leading to the high availability of skins worth valuing [14].

The yield of gelatin extraction was approximately 5%, per the literature for extractions from various fish skins under the same conditions [18,22,34,35,36,37].

### 3.2. Structural Characterization of Gelatin and GelMA

#### 3.2.1. FTIR Analysis

**Figure 2:**
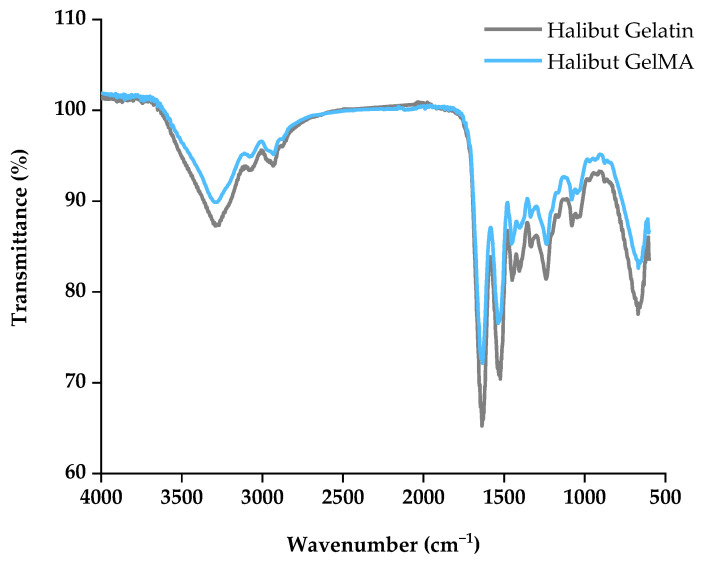
FTIR spectra of Greenland halibut gelatin (gray line) and derived GelMA (light blue line).

FTIR analysis can prove the existence of some chemical bonds of gelatin since this protein is usually portrayed by the presence of five signals of amide—amide A, amide B, amide I, amide II and amide III [38,39], as shown in the infrared spectrum of Greenland halibut skin gelatin (gray line) in Figure 2. Amide-A, which represents NH-stretching coupled with hydrogen bonding, appeared at 3271 cm^−1^, suggesting the hydrogen bonding involvement of an NH group in α-chain since normally, a free NH stretching vibration is found in the range of 3400–3440 cm^−1^ [40]. Amide-B was observed at 3064 cm^−1^, associated with NH stretching vibration. Another important peak was observed at 2924 cm^−1^ (asymmetrical), representing CH stretching vibrations of the -CH_2_ groups [39,41]. Amide I signal appeared at the wavenumber of 1637 cm^−1,^ which results from C=O stretching vibration from the amide group on proteins [39]. Among these absorption bands, the amide I band between 1600 and 1700 cm^−1^ is the most useful peak for infrared analysis of the secondary structure of proteins like gelatin [42]. The amide II band was observed at 1523 cm^−1^, attributed to an out-of-phase combination of C=N and C=C stretch and in-plane NH deformation modes of the peptide group. Other important peaks observed at 1450, 1406, and 1330 cm^−1^ are absorptions arising from wagging vibrations from CH_2_ groups from the glycine backbone and proline side chains [41,43]. Amide III represents the combination peaks between C=N stretching vibrations and NH deformation from amide linkages observed at 1240 cm^−1^. Other important peaks associated with the C=O stretching vibrations of the short peptide chains were observed at 1080 and 1031 cm^−1^ [39,40,43].

FTIR analysis was also performed with GelMA samples (Figure 2, light blue line). The GelMA spectrum showed a similar pattern compared with the halibut gelatin spectrum. Although methacrylate substituent groups were incorporated into the gelatin, this was not detectable by FTIR spectra [28,44]. This can be because the chemical modification of gelatin by methacrylic anhydride normally requires less than 5% of the amino acid residues in molar ratio, meaning that there was no significant impact on most of the functional amino acid motifs, such as the arginine-glycine-aspartic acid sequence (RGD) and metalloproteinase degradable motifs [45].

#### 3.2.2. CD Analysis

**Figure 3:**
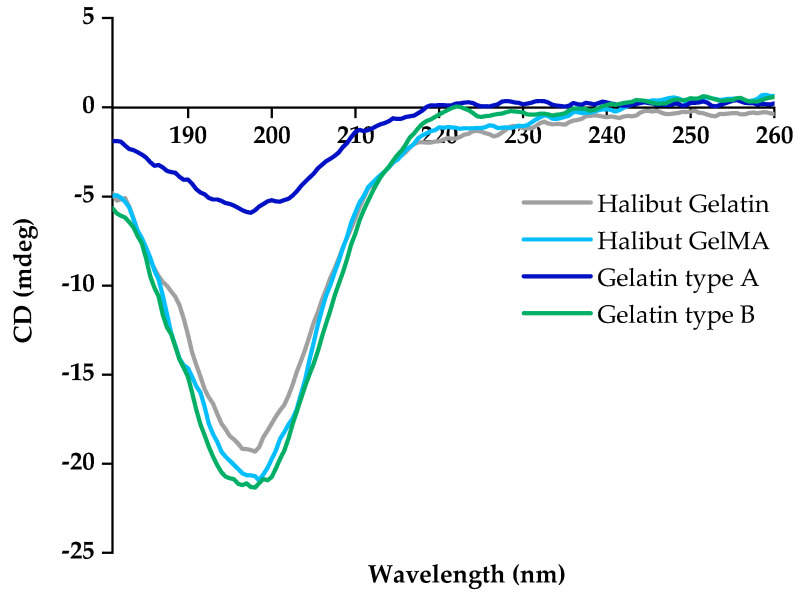
CD spectra of Greenland halibut skin gelatin (gray line), derived GelMA (light blue line), commercial gelatin type A (dark blue line) and commercial gelatin type B (green line).

The protein conformation of halibut gelatin was evaluated through CD analysis. The obtained spectrum was consistent with the presence of random coil conformation of gelatin (Figure 3, gray line). Compared with type A and type B commercial gelatins, the results showed that gelatin extracted from Greenland halibut skin has an identical profile to commercial type B gelatin (Figure 3, green line) [46]. In the present study, the CD spectrum of gelatin exhibits a marked decrease in the negative intensity around 200 nm, which is characteristic of proteins with a random coil [47,48,49]. In addition, the slight CD-peak around 220 nm appears in the negative part of the graph, which could indicate the development of chain reversals in the peptide chains after the destruction of the triple helical structure [23,50].

CD analysis was also performed with GelMA samples (Figure 3, light blue line). As in the FTIR analysis, it was also obtained almost identical CD spectra of unmodified gelatin and gelatin-methacryloyl demonstrated that functionalization did not induce a conformational change in the gelatin.

#### 3.2.3. NMR Analysis and DoF

**Figure 4:**
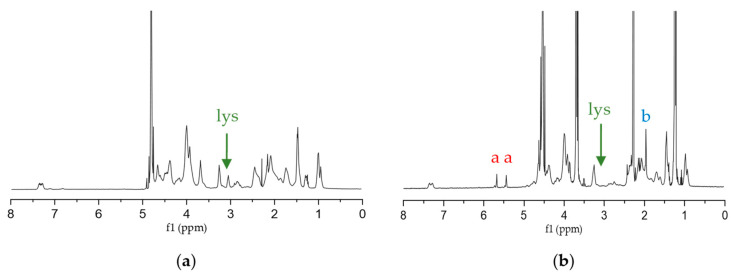
NMR spectra of (**a**) Greenland halibut gelatin; (**b**) Greenland halibut GelMA. “lys” in green corresponds to lysine methylene proton. Protons “a” in red and “b” in blue are associated with the methacryloyl group, as represented in the chemical structure of GelMA in Figure 1.

^1^H NMR spectra of non-methacrylated and methacrylated gelatin are shown in Figure 4a,b, respectively. Comparing both spectra, it is possible to observe two peaks at 5.4 and 5.6 ppm (peak “a” in Figure 4b) in GelMA spectra, nonexistent in the gelatin spectra, that match the two protons of methacryloyl double bond [16]. It proved that methacrylic anhydride was efficient in the functionalization of gelatin [5,25,28,51,52,53]. The new signal around 1.9 ppm (peak “b” in Figure 4b) corresponds to the methyl group present in the grafted methacryloyl [28,52,54]. Moreover, the peak at 3.0 ppm that is assigned to lysine (“lys”, Figure 4) [5,28,54] is much smaller in the GelMA spectrum when compared to the gelatin spectrum. A high degree of functionalization (DoF) means that less amines are free because they have been occupied with methacryloyl groups, and therefore, less free lysine is present. As such, the DoF can be quantified by integration (Equation (2)). In this work, DoF was calculated as approximately 58%. Since it influences the cross-linking density and the porosity and mechanical properties of hydrogel constructs, it needs to be adjusted based on the cell type used [11]. If, on the one hand, the increase of the DoF leads to stiffer hydrogels with a higher cross-linking density and improved shape, on the other hand, it decreases their pore size and permeability [11]. Furthermore, decreasing the DoF (less than 20%) causes problems handling the produced hydrogels (after photocrosslinking) during the various processes of evaluating their physical and mechanical properties. Due to that, low DoF GelMA requires much longer under UV light to form hydrogels, which is not viable when having hydrogels with encapsulated cells [54].

### 3.3. Characterization of GelMA Hydrogels

Photo-crosslinking is a widely used method to produce hydrogels for tissue engineering. GelMA is a good option for that process since it undergoes rapid photo-crosslinking due to the methacrylation of amino and carboxyl groups on the gelatin side chain. In addition, it conserves the RGD sequence and enzymatic degradability of gelatin’s cell interaction-promoting groups, which enables improved cell adhesion, promotes cell growth and proliferation, and regulates cell viability [55]. Pure GelMA hydrogels, however, have poor mechanical properties and a relatively fast degradation rate [56,57]. For this reason, mixing GelMA with GAGs such as hyaluronic acid and chondroitin sulfate (the most commonly found in the human body) is highly beneficial [58].

**Figure 5:**
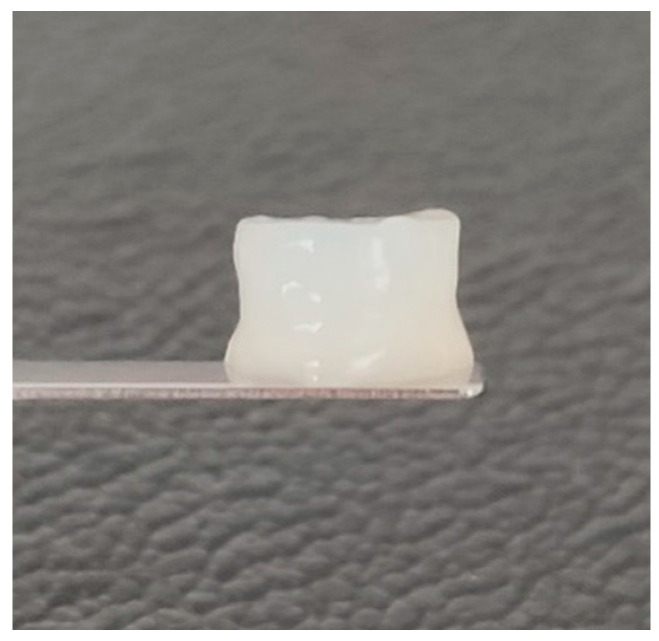
Hydrogel of formulation L (70% GelMA, 15% CSMA, 15% HAMA). An illustrative example of the produced hydrogels.

GelMA, derived from collagen type I, was the main component in all the hydrogels used in this study (Figure 5), accounting for at least 70% of the polymer content by mass. Figure 5 shows that hydrogels do not show high transparency, which is not an important feature in this type of study.

#### 3.3.1. Water Absorption Capacity

**Figure 6:**
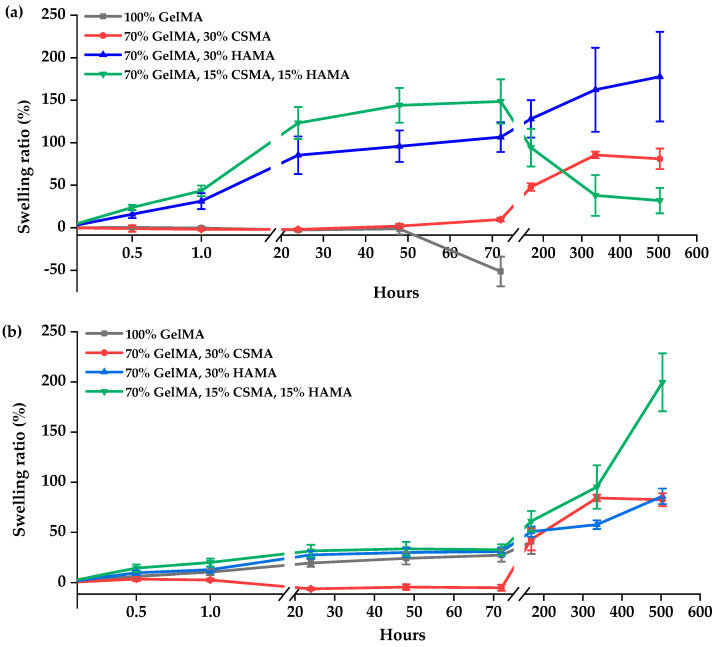
Swelling ratio (%) of hydrogels produced with (**a**) 10% (*w*/*v*) GelMA or; (**b**) 20% (*w*/*v*) GelMA upon incubation in PBS.

The determination of swelling represents an important aspect of the characterization of polymeric materials intended for biomedical applications, as it influences the capacity for oxygen and nutrient transfer within scaffolds [52]. Therefore, the swelling behavior of GelMA and GelMA/GAG hydrogels was investigated, and the main observations are shown in Figure 6. The results for hydrogels produced with 5% (*w*/*v*) GelMA (samples A, B, C and D) were inconclusive. The swelling tests did not show absorption capacity, and the hydrogels degraded after 24 h, which may have happened because they were not sufficiently cohesive. These results could be related to decreasing the polymer concentration generally producing softer/viscous hydrogels [11], and photocrosslinking was not sufficiently extensive. Therefore, no further tests were performed on hydrogels produced with 5% (*w*/*v*) GelMA.

For most hydrogels prepared with GelMA solutions at 10% and 20% (*w*/*v*) concentrations, the swelling ratios increased over time until they stayed constant. However, some hydrogels did not show an initial increase, such as E (Figure 6a, gray line) and F (Figure 6a, red line), and, in the case of the formulation H (Figure 6a, green line), there was a dramatic decrease after 72 h, which may have been due to intramolecular hydrogen bonding, which restricted the hydrogel swelling [52,53]. Other hydrogels, such as hydrogels G (Figure 6a, blue line) and L (Figure 6b, green line), showed a higher viscosity and, therefore, more difficulty in handling, which is directly related to higher percentages of water uptake. At the end of the 21 days (504 h), comparing the results of hydrogels produced with 10% (*w*/*v*) and 20% (*w*/*v*) GelMA, in general, the hydrogels with a higher concentration of GelMA showed a higher percentage of water uptake. Additionally, all formulations with GAG exhibited a higher swelling ratio than the bare GelMA hydrogels. Therefore, the addition of CSMA and HAMA to GelMA hydrogels led to an increase in the hydrophilicity of hydrogels, which means that they might be able to stimulate cell migration and facilitate the transport of nutrients necessary for cell growth within scaffolds [59].

#### 3.3.2. Enzymatic Degradation Capacity

**Figure 7:**
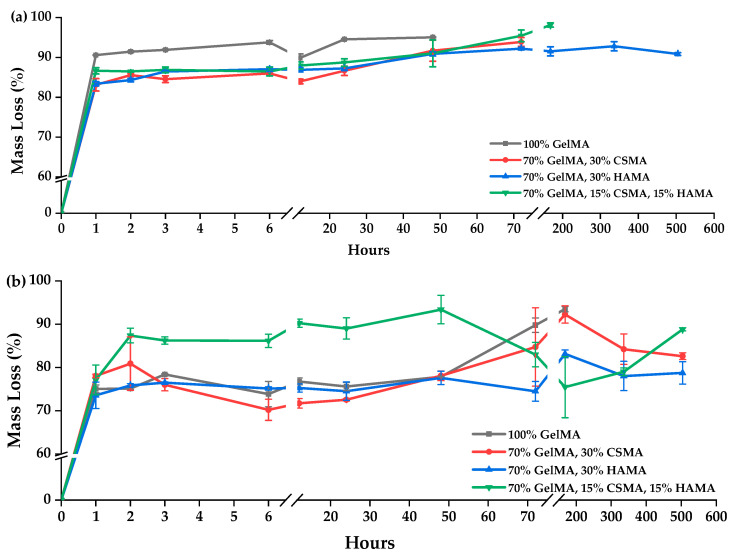
Mass Loss ratio (%) of hydrogels produced with (**a**) 10% (*w*/*v*) GelMA solution or; (**b**) 20% (*w*/*v*) GelMA solution, upon incubation in PBS with collagenase.

It is important to investigate the biodegradability of hydrogels before their biomedical applications to confirm if GelMA is susceptible to enzymatic degradation and that the developed hydrogels would act as temporary ECM surrogates once implanted, supporting new tissue formation but be degraded meanwhile and eliminated afterwards, as aimed for a tissue engineering scaffold. In this sense, the enzymatic degradability of hydrogels was analyzed in the presence of collagenase. Collagenase is a member of the matrix of the metalloproteinase family than can degrade and remodel the ECM for cell spreading and migration [5]. As expected, the hydrogels degraded in the presence of collagenase (Figure 7), indicating that GelMA-based materials will also be susceptible to local degradation via cell-secreted enzymes. As shown, hydrogel degradation is faster for a lower concentration of GelMA, with formulation E (Figure 7a, gray line) showing complete degradation in 2 days (48 h) and formulation I (Figure 7b, gray line) showing complete degradation in 7 days (168 h). These formulations are the ones that only had GelMA in their composition, and these results were according to the literature [5,13,54,60]. For 10% (*w*/*v*) GelMA solution hydrogels, similarly to the findings for swelling capacity, formulation G (Figure 7a, blue line) obtained the best results. The remaining ones, namely F (Figure 7a, red line) and H (Figure 7a, green line), degraded completely after 3 days (72 h) and 7 days (168 h), respectively. For 20% (*w*/*v*) GelMA solution-based formulations, formulation L (Figure 7b, green line) also showed a dramatic decrease, which can again be explained by a possible increase in hydrogen bonding.

### 3.4. Biological Assessment

Tissue engineering scaffolds cultured with cells can provide a more direct approach to promote cartilage tissue regeneration. Modular tissue engineering holds great potential in regenerating natural complex tissues by engineering three-dimensional modular scaffolds with predefined geometry. In modular tissue-like construction, a scaffold with high biocompatibility for cell survival is the key to successful bioconstruction [61]. In this work, different composite hydrogels based on a combination of GelMA and CSMA/HAMA were exploited, with combinations of these being done to promote cell functional expression for cartilage tissue engineering inspired by the composition of the native cartilage ECM. The GelMA concentration chosen was 20% (*w*/*v*), not only because it showed good results in water absorption and enzymatic degradation but also because it produced more cohesive hydrogels and, therefore, was easier to handle.

#### 3.4.1. Live/Dead Assay

**Figure 8:**
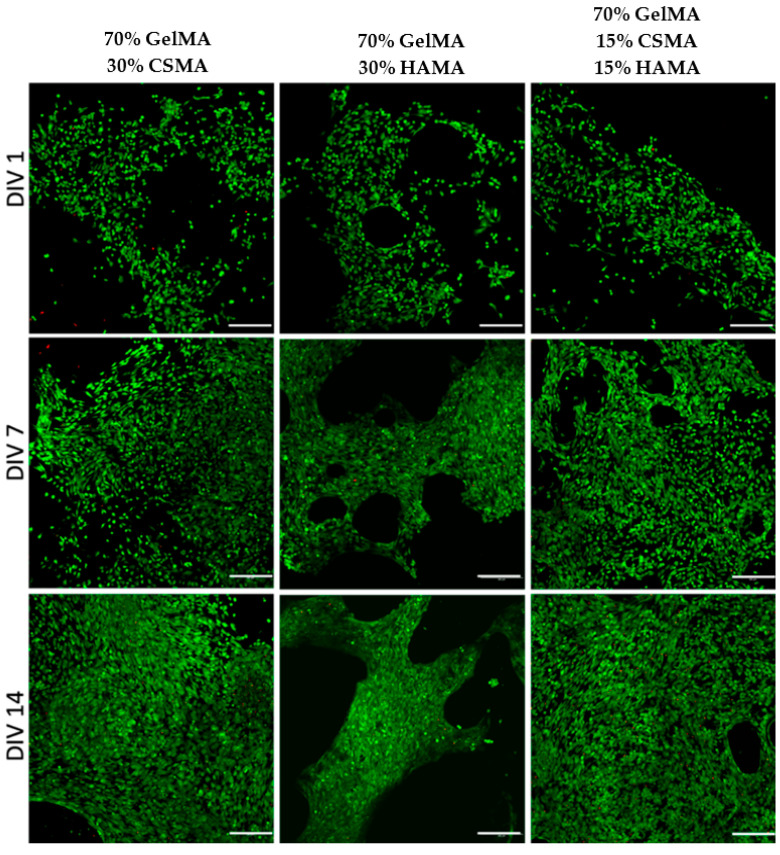
Confocal images of live (green)/dead (red) assay for the ATDC-5 cells seeded on the different GelMA-based hydrogels at 1, 7 and 14 days in culture. The bar represented 200 µm.

Chondrogenic cells were seeded in GelMA-based hydrogels combined with CSMA or/and HAMA. It was possible to observe that cellular density increased with in vitro culture time for all the hydrogel compositions (Figure 8). Although the ATDC-5 cells have been seeded on top of the hydrogels and not encapsulated, it was considered a 3D cell culture, given the nature of the hydrogels and the possibility for cells to migrate inside them, depending on the material-cell interaction. A 2D culture would be the case if the seeded were performed onto an “impenetrable” surface, such as the bottom of microplate wells or metallic surfaces.

The live/dead assay indicated that cells remained viable for up to 14 days in culture and showed a successful distribution of the cells in all GelMA hydrogel formulations, as could be observed by the green signal assignment. In general, for all formulations, it is possible to see that. Initially, the cells had a round shape and were spread across the hydrogel but over time, the number of cells with an elongated shape increased. These qualitative results indicate that all the biomaterials’ conditions allowed the adhesion of chondrogenic cells without significant macroscopic differences observed, in agreement with the results observed by others with GelMA/GAGs templates for the cell culture [1].

Other studies using materials containing GelMA [62,63,64] obtained similar results in live/dead assays, confirming that GelMA-based scaffolds can provide a favorable environment for cartilage-like matrix deposition.

#### 3.4.2. Analysis of Metabolic Activity and Proliferation

**Figure 9:**
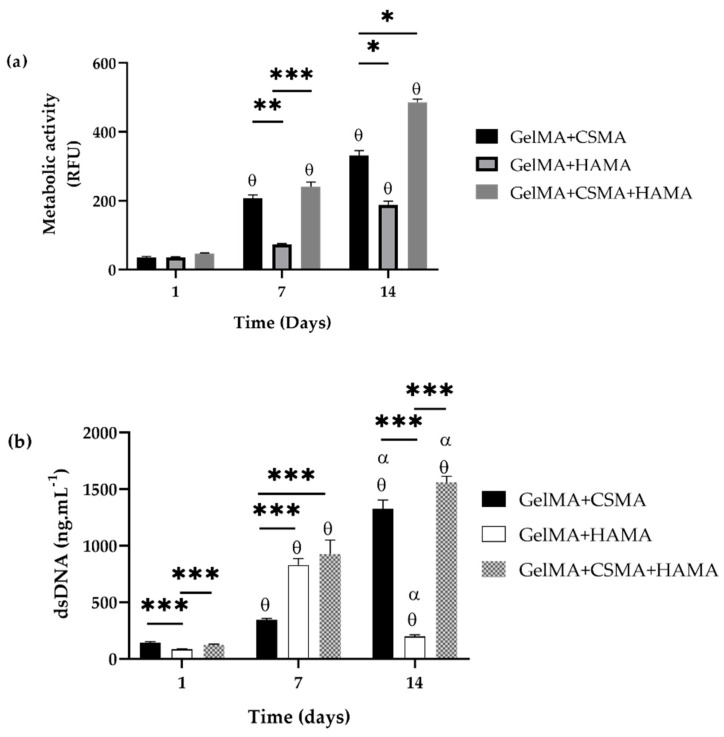
In vitro assessment of the biological performance of different GelMA-based hydrogels seeded with ATDC5 over cell culture time, with the correspondent statistical analysis (* *p* < 0.05, ** *p* < 0.01, *** *p* < 0.001, and symbols denote statistical differences when compared each of the conditions at each time point and compared with day 1 (θ) and day 7 (α). Data are presented as mean ± SD (*n* = 3). (**a**) ATDC5 metabolic activity; (**b**) ATDC5 proliferation assessed by quantification of dsDNA.

The cytotoxicity of the GelMA-based hydrogels was assessed by evaluating the metabolic activity of ATDC-5 cells seeded on the hydrogels, and the obtained results are collated in Figure 9a. For all tested hydrogels, the metabolic activity of the ATDC-5 cells increased with time in the culture. When comparing the different GelMA-based hydrogels, the results are quite similar over time in culture, and all three different formulations show an increase in metabolic activity. Furthermore, the formulation with CSMA and HAMA (simultaneously) stands out, with the highest metabolic activity on day 14. These results confirmed the cytocompatibility of the materials used and demonstrated that their biological activity wasn’t compromised by the production methodology.

Analyzing the results obtained for the dsDNA quantification tests (Figure 9b), it was possible to verify that the cells proliferated over time, except for the GelMA/HAMA formulation, which had a sharp decrease after 7 days. This may be related to some alteration in the morphology of the hydrogel.

In general, the results agree with the findings from other researchers also studying hydrogels containing combinations of GelMA/GAGs [1] and with studies that include hydrogels containing GelMA [65,66]. However, Levett et al. [1] state that concentrations higher than 5% of HAMA content by mass may not affect chondrogenesis or even decrease, which was not verified in this study. HAMA also contributed to promoting chondrocyte phenotype and improving matrix distribution.

The previously mentioned Increase in green signal intensity throughout the cell culture time, together with the increment of metabolic activity and proven proliferative state of the cells, seems to prove that these biomaterials can offer an adequate structural environment for hosting chondroblasts cells and can be used for cartilage tissue engineering. These results may be related to the biological recognition sites on gelatin, including RGD sequences, which induce cell adhesion and proliferation [66].

#### 3.4.3. SEM Analysis

**Figure 10:**
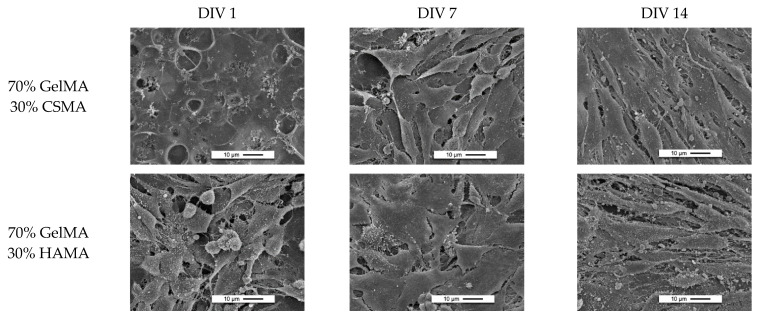

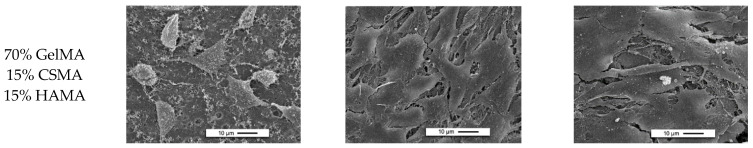
SEM images obtained of 20% (*w*/*v*) GelMA solution-based hydrogels seeded with ATDC5 cells at ×1.500 magnification.

SEM imaging was performed with the ATDC5-coated hydrogels after 1, 7 and 14 days of incubation (Figure 10). Cells can change their size and shape based on their environment [67]; thus, differences in observed cell morphology indicate differences between the various hydrogels. After analyzing the SEM images, a general tendency of the cells to be round was observed. It was especially observed in hydrogels with HAMA [1,45]. The rounded morphology observed in healthy cartilage appears intrinsically linked to cell phenotype [68]. When placed on two-dimensional surfaces coated with ECM molecules, sub-populations of rounded and spread cells can be observed [69]. While rounded cells continue producing predominantly collagen type II, spread cells produce collagen type I and fibronectin [69], suggesting that reacquiring a rounded morphology may be a requirement for redifferentiation.

In general, it was possible to see the existence of smaller pores, which was corroborated by the fact that higher DoF values of GelMA lead to stiffer hydrogels, whereas the pore size decreases. This structural characteristic may be the reason for the visible lower number of cells in each of the 20% (*w*/*v*) GelMA solution formulations [70]. Even so, it could be seen that the cells begin to form their extracellular matrices over time. This, again, proves that the developed 3D scaffolds containing a mixture of GelMA/GAGs are a good biomaterial to host chondroblasts cells.

## 4. Conclusions

This research focused on developing a bioartificial construct capable of mimicking the cartilage tissue characteristics and providing the ideal environment for effective integration and repair. Among the natural polymeric materials, fish gelatin offers great practical potential as a scaffolding material due to its properties like biocompatibility, biodegradability, and good mechanical properties. In this regard, fish gelatin was extracted from the skins of Greenland halibut, thus offering the possibility for the valorization of this fish processing by-product. Regarding its application in biomaterials and to further improve intrinsic characteristics, fish gelatin was successfully modified via one-step chemical methacrylation, enabling photocrosslinking. This enabled the production of hybrid hydrogels based on GelMA and GAGs, namely CS and HA. Functionalization with methacryloyl moieties resulted in more cohesive matrices with improved water absorption capacity and biodegradability.

Furthermore, the GelMA-based hydrogels prepared, which can be easily synthesized in the lab for a low price, promoted adhesion, spread, and proliferation of cells, mimicking the structure of native tissues. From the selected formulations (all based on the 20% (*w*/*v*) GelMA solution), the L formulation (70% GelMA, 15% CSMA, 15% HAMA) should be highlighted, especially because, in addition to leading to more cohesive hydrogels, it still showed good cellular performance, i.e., the cells seeded on the hydrogel scaffold have demonstrated the ability to adhere and proliferate. Nevertheless, the time needed to promote effective photocrosslinking is a shortcoming of the present approach regarding the possibility of cell encapsulation, as encapsulated cells may be affected by such exposure, namely regarding DNA damage. Thus, future studies should address the tuning of the proposed hydrogels, varying the GelMA concentration, methacrylation degree and, consequently, the crosslinking extension, aiming to establish a shorter (cell-friendly) UV irradiation while assessing the effect over hydrogel stiffness. Likewise, the impact on the maintenance of chondrogenic phenotype and expression of extracellular matrix components towards new cartilage tissue formation should also be confirmed.

## Figures and Tables

**Table 1:** Composition and notation of produced GelMA-based hydrogels.

The Concentration of GelMA Solution (*w*/*v*)	Biopolymers Ratio (*w*/*w*)	Notation
5%	100% GelMA	A
	70% GelMA, 30% CSMA	B
	70% GelMA, 30% HAMA	C
	70% GelMA, 15% CSMA, 15% HAMA	D
10%	100% GelMA	E
	70% GelMA, 30% CSMA	F
	70% GelMA, 30% HAMA	G
	70% GelMA, 15% CSMA, 15% HAMA	H
20%	100% GelMA	I
	70% GelMA, 30% CSMA	J
	70% GelMA, 30% HAMA	K
	70% GelMA, 15% CSMA, 15% HAMA	L

## Data Availability

The data that support the findings of this study are available from the corresponding author, upon reasonable request.
